# Pilot study of cerebrospinal fluid biomarkers reveals inflammatory changes in patients with paranoid schizophrenia

**DOI:** 10.1038/s41598-025-13367-8

**Published:** 2025-08-03

**Authors:** Franz Felix Konen, Philipp Sebastian Gehring, Hannah Benedictine Maier, Sebastian Schröder, Seda Nur Türker, Helge Frieling, Stefan Bleich, André Huss, Hayrettin Tumani, Daniel Lüdecke, Jürgen Gallinat, Berend Malchow, Niels Hansen, Jens Wiltfang, Alexandra Neyazi, Thomas Skripuletz, CAP (Cerebrospinal Fluid Analysis in Psychiatry) Consortium, Franz Felix Konen, Hannah Benedictine Maier, Helge Frieling, Stefan Bleich, Daniel Lüdecke, Jürgen Gallinat, Niels Hansen, Jens Wiltfang, Alexandra Neyazi, Thomas Skripuletz

**Affiliations:** 1 Department of Neurology, Hannover Medical School, Carl-Neuberg-Straße 1, 30625 Hannover, Germany; 2 Department of Psychiatry, Social Psychiatry and Psychotherapy, Hannover Medical School, Carl-Neuberg-Straße 1, 30625 Hannover, Germany; 3 Laboratory for Molecular Neuroscience, Department of Psychiatry, Social Psychiatry and Psychotherapy, Hannover Medical School, Carl-Neuberg-Str. 1, 30625 Hannover, Germany; 4 Department of Neurology, University Hospital Ulm, Oberer Eselsberg 45, 89081 Ulm, Germany; 5 Department of Neurology, CSF Laboratory, University Hospital Ulm, Oberer Eselsberg 45, 89081 Ulm, Germany; 6 Department of Psychiatry and Psychotherapy, University Hospital Hamburg-Eppendorf, Martinistraße 52, 20246 Hamburg, Germany; 7 Department of Psychiatry and Psychotherapy, University Medical Center Goettingen, Von-Siebold-Str. 5, 37075 Goettingen, Germany; 8 German Center for Neurodegenerative Diseases (DZNE), Von-Siebold-Straße 3A, 37075 Göttingen, Germany; 9 Neurosciences and Signaling Group, Department of Medical Sciences, Institute of Biomedicine (iBiMED), University of Aveiro, Aveiro, 3810-193 Portugal; 10 Department of Psychiatry and Psychotherapy, Otto-von-Guericke-University Magdeburg, Leipziger Str. 44, 39120 Magdeburg, Germany

**Keywords:** Paranoid schizophrenia, Cerebrospinal fluid, Kappa free light chains, Neurofilament light chains, Cytokines, Chemokines, Biomarkers, Psychosis, Schizophrenia

## Abstract

Paranoid schizophrenia is a severe mental illness with both positive and negative symptoms. Currently, the role of peripheral and central inflammation is increasingly suspected as possible factor in the pathogenesis of schizophrenia. This retrospective, monocentric pilot study investigated 35 patients (15/35 female) diagnosed with paranoid schizophrenia after exclusion of possible underlying neuroinflammatory disorders to assess for inflammatory changes of the cerebrospinal fluid (CSF) and associated signs of neurodegeneration. Kappa free light chains (KFLC), a panel of 21 cyto- and chemokines, and neurofilament light chains (NFL) as surrogate parameters for neuro-inflammation and -degeneration were determined in patients with paranoid schizophrenia as well as age- and sex-matched inflammatory (*n* = 35) and non-inflammatory controls (*n* = 40). Patients with paranoid schizophrenia exhibited significantly higher intrathecal synthesized fractions of KFLC than non-inflammatory controls. KFLC-positive patients with paranoid schizophrenia had significantly higher NFL concentrations in CSF than KFLC-negative patients according to Reiber´s diagram. NFL concentrations in CSF of patients with paranoid schizophrenia were associated with illness duration, frequency of psychotic episodes, and amount of antipsychotic treatment attempts. This pilot study highlights inflammatory changes in the CSF among a specific subgroup of patients with paranoid schizophrenia, positively correlating with elevated NFL levels in CSF.

## Introduction

Schizophrenia is a complex, severe, and heterogeneous mental illness characterized by positive (mostly auditory hallucinations) and negative symptoms (such as decreased emotional expression) as well as cognitive impairment^1^. Despite extensive research, pathogenesis of schizophrenia is not fully understood and no biomarkers have yet been implemented in clinical practice. To date, two rather complementary models of pathogenesis are discussed. First, there is the neurodevelopmental hypothesis, which proposes genetic and environmental factors as a major influence^2^. Based on various neuroimaging parameters, it has been proposed that brain changes during childhood and adolescence might contribute to the development of schizophrenia^2–4^. On the other hand, the early onset and steadily progressive course of cognitive decline in patients with schizophrenia was discussed more than 100 years ago as “dementia praecox,” a neurodegenerative disorder^5^. Accordingly, several neuroimaging and biomarker studies reported similarities between patients with schizophrenia and different types of dementias^6–8^. In addition to neurodegeneration, signs of inflammatory activity have also been described in schizophrenia, similar to early stages of Alzheimer´s disease^9^. Some studies found signs of inflammation in cerebrospinal fluid (CSF) such as an increase in inflammatory cells, immunoglobulins, cytokines and chemokines^10–14^. However, inflammation in schizophrenia is a complex and multifaceted issue and the literature is rich with conflicting results and varied methodologies^15^. Numerous studies have explored inflammation in schizophrenia through different lenses, such as immune dysregulation, neuroinflammation, and peripheral inflammatory markers like cytokines^15^. In these investigations, various immune-related pathways including microglial activation and blood-brain barrier dysfunction have been reported and a large diversity of inflammatory markers investigated^10–15^. However, in the past years markers for neuro-axonal damage in patients diagnosed with neuroinflammatory diseases emerged as potential diagnostic and prognostic markers^16^. Recently, neurofilament light chains (NFL), which form crucial intermediate filaments for neuron assembly, have been investigated to distinguish dementias and autoimmune mediated encephalitis from primary psychiatric disorders^16–20^. Here, NFL was highlightened as exclusionary biomarker and it was concluded that NFL do not hold diagnostic value for patients with psychotic disorders^16–20^. Nevertheless, NFL are known to be involved in the regulation of neurotransmission and synapses, thus NFL have been investigated in serum and CSF of patients with psychotic disorders to analyze their utility to assess for prognosis and diagnosis in specific subgroups of patients with psychotic disorders^21–24^. In these studies, NFL revealed usefulness as potential biomarker for illness progression and treatment resistance in schizophrenia^21–24^.

Kappa free light chains (KFLC), a byproduct of intact immunoglobulin synthesis by B and plasma cells, have become established as an additional diagnostic parameter for the detection of the humoral immune response in the CNS, alongside the gold standard, oligoclonal bands^25^. Although KFLC have been shown to detect intrathecal immunoglobulin synthesis with high accuracy in patients with various inflammatory and non-inflammatory neurological disorders, they have not yet been studied in patients with schizophrenia^26^.

Therefore, the aim of the present study was to assess for inflammatory changes (KFLC, cyto-/chemokines) and inflammation-related neurodegeneration (NFL) in the CSF of patients with paranoid schizophrenia thus uniting the currently discussed pathophysiological models of neuroinflammation and neurodegeneration.

## Results

### Patients

Patient’s characteristics are shown in Table 1. The sex distribution of the patients with paranoid schizophrenia was similar and 15/35 (43%) of the included patients were female, while 20/35 (57%) were male. Participants all self-identified as white Caucasians. The mean age at lumbar puncture was 30 years. The control subjects with inflammatory and non-inflammatory diseases were similar in sex (15/35 and 25/40 females, *p* = 0.336) and age (30 and 35 years, *p* = 0.096).

**Table 1:** Characteristics of patients with schizophrenia at admission.

Demographics	
Females / males	15/20
Age [years], median (interquartile range)	30 (23–40)
Amount of psychotic episodes [n], median (interquartile range)	2 (2–9)
First psychotic episode, n (%)	12/35 (34%)
Second psychotic episode, n (%)	9/35 (26%)
3 or more psychotic episodes, n (%)	14/35 (40%)
Time between symptom onset and LP [months], median (interquartile range)	38 (7-118)
**Substance users**	
Smoker (at least 12 months), n (%)	25/35 (71%)
Alcohol use disorder diagnosis, n (%)	8/35 (23%)
Cannabis (at least 12 months), n (%)	8/35 (23%)
Other drugs, n (%)	5/35 (14%)
**Clinial presentation of schizophrenia**	
Delusions / without delusions [n]	20/15
Hallucination / without hallucinations [n]	19/16
Assured disturbance of self-experience / without assured disturbance of self-experience [n]*	10/25
Disorganization / without disorganization [n]	31/4
Psychomotor drive reduced / psychomotor drive increased [n]	24/11
Suicidal ideation / without suicidal ideation [n]	6/29
Amount of suicide attempts [n], median (min-max)	0 (0–5)
**Treatment**	
Antipsychotic pharmaco-therapy, n (%)	28/35 (80%)
First anti-psychotic pharmaco-therapy attempt, n (%)	13/28 (46%)
Second anti-psychotic pharmaco-therapy attempt, n (%)	3/28 (11%)
3 or more anti-psychotic pharmaco-therapy attempts, n (%)	12/28 (43%)
Without antipsychotic pharmaco-therapy, n (%)	7/35 (20%)
**Antipsychotics at sampling**,** n (%)**	28/35 (80%)
Typical antipsychotic with high potency, n (%)	15/35 (43%)
Typical antipsychotic with low potency, n (%)	5/35 (14%)
Atypical antipsychotics, n (%)	18/35 (51%)
Clozapine, n (%)	2/18 (11%)
Olanzapine, n (%)	6/18 (33%)
**Antidepressants at sampling**,** n (%)**	4/35 (11%)
SSRI, n (%)	1/35 (3%)
SSNRI, n (%)	1/35 (3%)
Mirtazapine, n (%)	2/35 (6%)
Tricyclics, n (%), Lithium, n (%)	0/35, 0/35
Benzodiazepines, n (%)	18/35 (51%)
Anticonvulsants, n (%)	3/35 (9%)

SSRI = selective serotonin reuptake inhibitor; SSNRI = selective serotonin noradrenalin reuptake inhibitor.

*”Assured” meaning that the disturbance of self-experience was objectively assessed and confirmed by an experienced psychiatrist.

34% of patients with paranoid schizophrenia had their first psychotic episode on admission, 26% of patients had their second episode and 40% of patients had at least 3 psychotic episodes. The median time from symptom onset to lumbar puncture was 38 months.

The interview at admission revealed that most of the included patients with paranoid schizophrenia had delusions, hallucinations, disorganization and a reduced psychomotor drive, whereas in the majority of the patients with schizophrenia ego disorders could not have been assuredly detected (Table 1).

Serological analysis to investigate possible autoimmune diseases revealed a borderline elevated ANA titre of 1:160 in one patient with paranoid schizophrenia, while the other serological tests (extractable nuclear antigen antibodies (ENA), rheumatoid factor, anti-neutrophil cytoplasmic antibodies (ANCA), anti-thyroid antibodies, antineuronal antibodies (NMDA, CASPR2, LGI1. GABA, AMPAR, DPPX; anti-yo, -hu, -ri, -amphiphysin, -CV2/CRMP-5, -ma1, -ma2, -GAD, -sox1) were negative.

Virological (measles, rubella, varicella zoster, herpes simplex, Ebstein-Barr-virus, cytomegalic virus) and bacteriological (borrelia, syphilis) tests showed an elevated antibody specific index of 1.6 for rubella virus in one patient with paranoid schizophrenia without a concomitant elevated CSF cell count, while all other patients had normal results.

### Basic CSF parameters

Table 2 provides an overview of the basic CSF parameters for the included patients and controls.

**Table 2:** Basic cerebrospinal fluid (CSF) parameters.

CSF results	Schizophrenia (*n* = 35)	Non-inflammatory controls (*n* = 40)	Inflammatory controls (*n* = 35)
Elevated cell count (> 4/µl), n (%)	4 (11%)	0 *p* = 0.043	28 (80%) *p* < 0.001
Elevated albumin CSF-serum quotient, n (%)	14 (40%)	10 (25%) *p* = 0.217	15 (43%) *p* = 1
CSF total protein (mg/l), mean (SD)	424 (138)	469 (163) *p* < 0.001	354 (129) *p* < 0.001
Elevated CSF lactate [mmol/l], n (%)	0	0 *p* = 1	13 (37%) *p* < 0.001
Intrathecally synthesized IgG according to Reiber´s diagram [%], n (%)	0	0 *p* = 1	23 (66%) *p* < 0.001
Intrathecally synthesized IgA according to Reiber´s diagram [%], n (%)	0	0 *p* = 1	3 (9%) *p* = 0.239
Intrathecally synthesized IgM according to Reiber´s diagram [%], n (%)	1 (3%)	0 *p* = 0.467	10 (29%) *p* = 0.003
CSF-specific oligoclonal bands, n (%)	4 (11%)	0 *p* = 0.043	35 (100%) *p* < 0.001

CSF = cerebrospinal fluid; Ig = immunoglobulin; SD = standard deviation. The presented p-values reflect the comparison between the corresponding control group (inflammatory or non-inflammatory) and individuals with schizophrenia.

In the majority of patients with paranoid schizophrenia, the basic CSF parameters were normal or showed non-specific changes, such as a blood-CSF barrier dysfunction (40%) as indicated by elevated albumin CSF-serum quotient. In four individual patients (11%), CSF pleocytosis and CSF-specific oligoclonal bands were observed including one patient who displayed isolated intrathecal IgM synthesis in Reiber’s diagram.

Patients diagnosed with MS exhibited inflammatory changes in the basic CSF parameters significantly more often than patients diagnosed with paranoid schizophrenia, including frequent CSF pleocytosis (80%), blood-CSF barrier dysfunction (43%), intrathecal immunoglobulin IgG (66%), IgA (9%) and IgM synthesis (10%), and CSF-specific oligoclonal bands (100%). Non-inflammatory controls were characterized by mainly normal CSF findings, without pleocytosis or intrathecal immunoglobulin synthesis. 25% of the non-inflammatory controls exhibited a blood-CSF barrier dysfunction. Comparison with patients with paranoid schizophrenia revelaed significant differences for the proportion of patients with pleocytosis and CSF-specific oligoclonal bands as well as the concentration of CSF total protein, which were significantly lower in non-inflammatory controls (Table 2).

### Kappa free light chains

Table 3 presents the results of KFLC measurements, acting as a surrogate marker for intrathecal inflammation, among the participants and controls. As described in the methods section, the Reiber diagram was applied to dichotomize whether an intrathecal synthesis of KFLC was present or not.

**Table 3:** Kappa free light chains (KFLC) and neurofilament light chain (NFL).

Cerebrospinal fluid (CSF) results	Schizophrenia (*n* = 35)	Non-inflammatory controls (*n* = 40)	Inflammatory controls (*n* = 35)
**KFLC**			
CSF KFLC concentration [mg/l], mean (SD)	0.2 (0.16)	0.2 (0.11) *p* = 0.260	6.93 (8.55) *p* < 0.001
Serum KFLC concentration [mg/l], mean (SD)	13.1 (5.54)	15.9 (5.49) *p* = 0.008	11.3 (4.45) *p* = 0.164
Intrathecal fraction (IF) > 0% according to Reiber´s diagram for KFLC, n (%)	7 (20%)	0 *p* = 0.003	35 (100%) *p* = 0.003
Intrathecal KFLC fraction according to Reiber´s diagram for KFLC [%], mean (SD)	33 (18.29)	0 *p* < 0.001	90.7 (9.08) *p* < 0.001
Intrathecally synthesized KFLC (KFLC_Loc_) [mg/l], mean (SD)	0.065 (0.13)	0.024 (0.04) *p* = 0.028	6.814 (8.56) *p* < 0.001
**NFL**			
CSF NFL concentration [pg/ml], mean (SD)	399 (220)	733 (1380) *p* = 0.119	1984 (2557) *p* < 0.001
Serum NFL concentration [pg/ml], mean (SD)	20 (25)	68 (210) *p* = 0.777	27 (17) *p* = 0.005

CSF = cerebrospinal fluid; KFLC = kappa free light chains; NFL = neurofilament light chain; SD = standard deviation. The presented p-values reflect the comparison between the corresponding control group (inflammatory or non-inflammatory) and individuals with schizophrenia.

Among the patients with paranoid schizophrenia, 20% exhibited intrathecal KFLC synthesis as per Reiber’s KFLC diagram, with an average synthesis rate of 33%. The average fraction of intrathecally synthesized KFLC was 0.065 mg/l. None of these patients with paranoid schizophrenia and intrathecal KFLC synthesis revealed abnormalities suggestive for seronegative autoimmune encephalitis or infections in MRI or EEG.

In comparison to individuals with paranoid schizophrenia, the control group of patients with inflammation (MS) showed significantly higher levels of KFLC in CSF (mean 6.93 mg/l vs. 0.2 mg/l). Additionally, these patients exhibited a higher prevalence of intrathecal KFLC synthesis according to Reiber’s diagram (100%), higher intrathecal synthesis (91%), and a larger proportion of intrathecally synthesized KFLC (6.8 mg/l).

In the comparison between non-inflammatory control individuals and patients with paranoid schizophrenia, it was evident that non-inflammatory control subjects exhibited significantly higher KFLC concentrations in their serum (mean 15.9 mg/l compared to 13.1 mg/l among patients with paranoid schizophrenia). Conversely, there was a significant difference in the percentage of patients with intrathecal KFLC synthesis according to Reiber’s diagram, with patients with paranoid schizophrenia showing a significantly higher proportion (33%) compared to the non-inflammatory control group (0%). Consequently, the rate of intrathecal synthesis (0%) and the fraction of KFLC synthesized intrathecally (0.024 mg/l) were lower among the non-inflammatory control subjects.

Since significantly higher KFLC levels were found in patients with paranoid schizophrenia compared with non-inflammatory controls, further characterization employing analyses of cytokines, chemokines, growth factors as well as NFL was enrolled.

### Cytokines, chemokines and growth factors

In terms of determination of cytokines and chemokines as well as growth factor concentrations in CSF, all samples revealed detectable concentrations of MCP-1, IL-6, IL-8, and sTREM-2 (Table 4). For all other cytokines and chemokines as well as growth factors, concentrations were detectable in less than 90% of the patients and were thus not suitable for statistical analyses.

**Table 4:** Cytokines, chemokines and growth factors in cerebrospinal fluid (CSF).

Cerebrospinal fluid (CSF) results	Schizophrenia (*n* = 35)	Non-inflammatory controls (*n* = 40)	Inflammatory controls (*n* = 35)
MCP-1 concentration [pg/ml], mean (SD)	650 (410)	667 (455) *p* = 0.862	316 (299) *p* < 0.001
IL-8 concentration [pg/ml], mean (SD)	48 (053)	51 (76) *p* = 0.602	178 (178) *p* = 0.002
sTREM-2 concentration [pg/ml], mean (SD)	4552 (5371)	4677 (4299) *p* = 0.384	4324 (3179) *p* = 0.529
IL-6 concentration [pg/ml], mean (SD)	2.68 (3.48)	3.38 (8.07) *p* = 0.751	1.61 (2.55) *p* = 0.215

CSF = cerebrospinal fluid; MCP-1 = monocyte chemoattractant protein 1; IL = interleukin; sTREM-2 = soluble triggering receptor expressed on myeloid cells; SD = standard deviation. The presented p-values reflect the comparison between the corresponding control group (inflammatory or non-inflammatory) and individuals with schizophrenia.

The concentrations of MCP1 were statistically significantly lower in patients diagnosed with MS compared to patients with paranoid schizophrenia. There were no significant differences between patients with paranoid schizophrenia and non-inflammatory controls.

The IL-8 concentrations were significantly higher in patients diagnosed with MS compared to patients with paranoid schizophrenia. There were no significant differences between patients with paranoid schizophrenia and non-inflammatory controls.

The concentrations of sTREM-2 were similar among the three groups. IL-6 concentrations tended to be slightly lower in patients diagnosed with MS compared to the other two groups, but the differences were not statistically significant.

### Neurofilament light chains

The concentrations of neurofilaments as surrogate parameter for neuro-axonal damage associated with intrathecal inflammation in both the CSF and serum were significantly higher in patients diagnosed with MS compared to patients with paranoid schizophrenia (Table 3; p-values < 0.001 and 0.005, respectively). No significant differences in neurofilament concentrations in CSF and blood were found between patients with paranoid schizophrenia and non-inflammatory controls (Table 3; p-values 0.119 and 0.777, respectively).

### Patients with paranoid schizophrenia with inflammatory characteristics

**Fig. 1:**
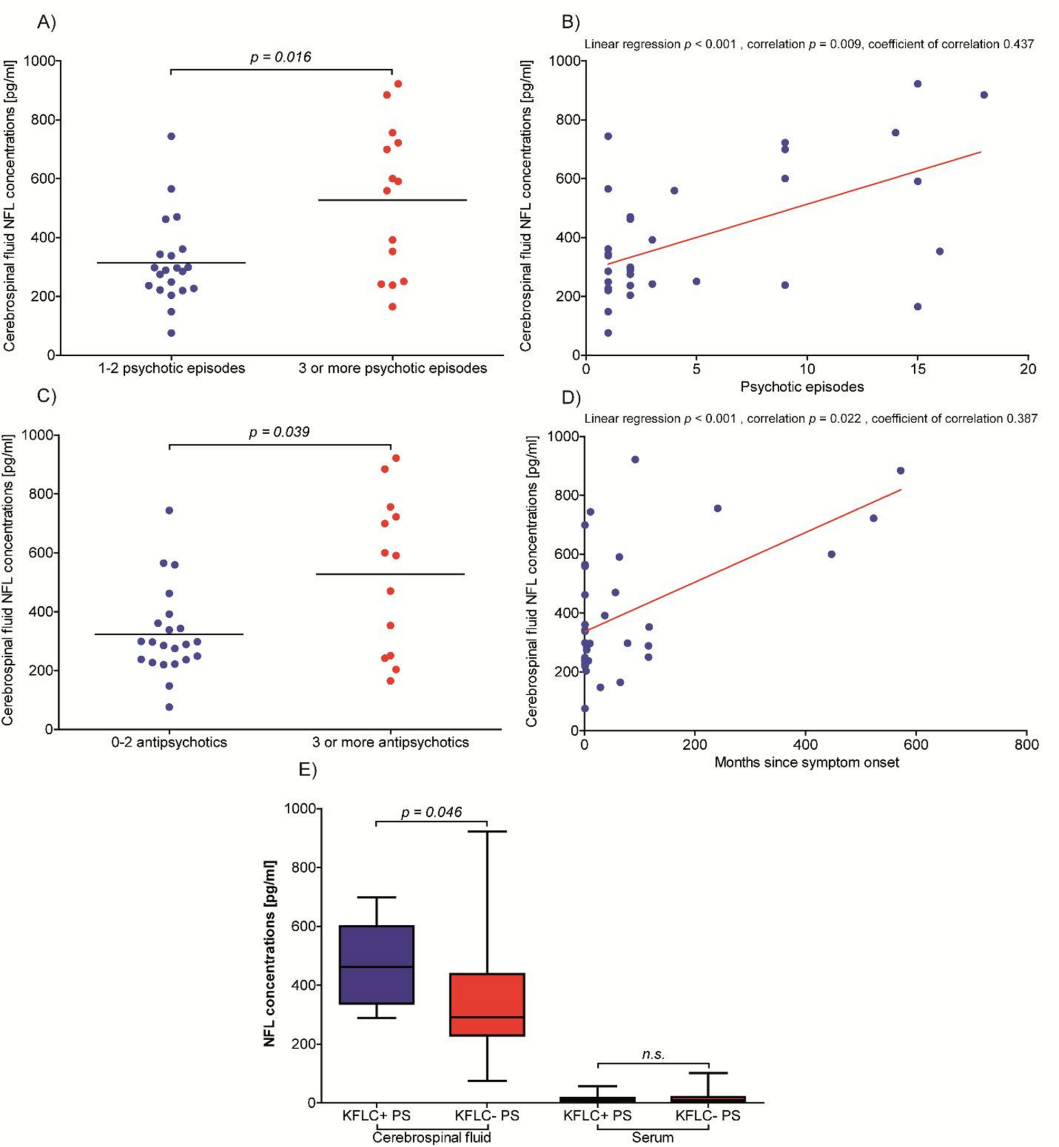
Neurofilament light chain (NFL) concentrations in patients with schizophrenia in association with different measures for illness severity. (**A**) CSF NFL concentrations in patients who experienced three or more psychotic episodes compared to patients in their initial or second episode. (**B**) Correlation between the number of psychotic episodes and CSF NFL concentrations. (**C**) CSF NFL concentrations in patients with a history of up to 2 antipsychotic treatment attempts compared to patients with 3 or more distinct antipsychotic treatment attempts. (**D**) Correlation between the number of months since symptom onset and CSF NFL concentrations. (**E**) CSF NFL concentrations in patients who exhibited intrathecal KFLC synthesis as indicated by Reiber’s diagram for KFLC compared to KFLC-negative patients. NFL = neurofilament light chain; KFLC = kappa free light chains; KFLC + = intrathecal KFLC synthesis according to Reiber´s diagram for KFLC; KFLC- = no intrathecal KFLC synthesis according to Reiber´s diagram for KFLC; ns = not significant (p-value > 0.05). P-values are indicated above the arrowed line or are given above the graph.

Patients with intrathecal KFLC synthesis according to Reiber’s diagram were compared with patients without intrathecal KFLC synthesis and thus without signs of inflammation in the CSF. Patients with paranoid schizophrenia who exhibited intrathecal KFLC synthesis as indicated by Reiber’s diagram for KFLC (referred to as KFLC + patients) demonstrated significantly higher NFL concentrations in CSF compared to KFLC-negative patients (459 pg/ml vs. 353 pg/ml, as depicted in Fig. 1E). The analysis of demographic, clinical, and laboratory parameters in KFLC + and KFLC- patients with paranoid schizophrenia did not yield any statistically significant differences (Table 5).

**Table 5:** Patients with schizophrenia with and without intrathecal KFLC synthesis.

Characteristics	Intrathecal KFLC synthesis (*n* = 7)	No intrathecal KFLC synthesis (*n* = 28)	*P*-value
Demographics			
Females / males	2/5	13/15	0.672
Age [years], median (interquartile range)	30 (29–56)	30 (22–39)	0.343
Months from symptom onset to LP, median (interquartile range)	118 (50–187)	18 (2–76)	0.073
**Clinical data**			
Delusions / without delusions [n]	2/7	18/28	0.112
Halluzination / without Halluzinations [n]	2/7	17/28	0.229
Assured disturbance of self-experience / without assured disturbance of self-experience [n]*	1/7	9/28	0.645
Psychomotor drive reduced / psychomotor drive increased [n]	5/7	19/28	1
Psychotic episodes [n], median (interquartile range)	2 (2–9)	2 (2–8)	0.395
Antipsychotics at sampling, n (%)	6 (86%)	22 (79%)	0.380
Laboratory parameters**			
NFL concentrations [pg/ml], mean (SD)	459 (149)	353 (200)	0.046
Serum NFL concentrations [pg/ml], mean (SD)	17 (18)	21 (27)	0.901
MCP-1 concentration [pg/ml], mean (SD)	619 (215)	658 (449)	0.757
IL-8 concentration [pg/ml], mean (SD)	48 (38)	48 (57)	0.695
sTREM-2 concentration [pg/ml], mean (SD)	8614 (9014)	3484 (3438)	0.224
IL-6 concentration [pg/ml], mean (SD)	2.84 (3.38)	2.64 (3.57)	0.635
eGFR [ml/min/1.73 m²], (SD)	105 (15)	108 (19)	0.703

CSF = cerebrospinal fluid; KFLC = kappa free light chains; MCP-1 = monocyte chemoattractant protein 1; IL = interleukin; sTREM-2 = soluble triggering receptor expressed on myeloid cells; SD = standard deviation.

*”assured” meaning that the disturbance of self-experience was objectively assessed and confirmed by an experienced psychiatrist.

**cerebrospinal fluid concentrations, if not otherwise stated.

### Association with parameters of illness severity in patients with paranoid schizophrenia

Since significantly different concentration levels of NFL in CSF between patients with paranoid schizophrenia with and without intrathecal KFLC synthesis were observed, different influencing factors were examined to explore their correlation with the severity of the condition within the group of patients with paranoid schizophrenia.

As an indicative measure of a persistent or ongoing illness course, the number of psychotic episodes up to the point of sampling was examined. Patients who experienced three or more psychotic episodes displayed significantly higher CSF NFL concentrations (527 pg/ml) compared to patients in their initial or second episode (315 pg/ml, *p* = 0.0161; Fig. 1A). Moreover, this group of patients also exhibited a significantly difference in age (median age for first/second episodes 26 years versus 43 years for ≥ 3 episodes; *p* = 0.0078), along with elevated serum KFLC levels (mean concentration for first/second episodes 11.6 mg/ml compared to ≥ 3 episodes 15.4 mg/ml; *p* = 0.0251). A multivariate analysis considering age as covariate was performed to verify the independence of the association between CSF NFL and the number of previous psychotic episodes. A significant linear regression and correlation emerged between the number of psychotic episodes and CSF NFL concentrations (Fig. 1B).

Another surrogate measure for a refractory illness course was the amount of antipsychotic treatment attempts. Patients with a history of up to 2 antipsychotic treatment attempts during the current psychotic episode exhibited significantly lower CSF NFL concentrations (324 pg/ml) compared to patients with 3 or more distinct antipsychotic treatment attempts (528 pg/ml; *p* = 0.0389; Fig. 1C). Antipsychotic drugs with anti-inflammatory properties (olanzapine, clozapine) were not significantly different used in patients with paranoid schizophrenia.

Lastly, the period from onset of symptoms to the time of sampling, serving as an indicator of a chronic illness course, was examined. After accounting for age and eGFR, a significant linear regression and positive correlation were identified between the number of months since symptom onset and CSF NFL concentrations (Fig. 1D).

Investigating KFLC concentrations in both CSF and serum, along with neurofilament concentrations in serum, as well as levels of cyto-, chemokines, and growth factors (MCP-1, IL-6, IL-8, and sTREM-2), no significant differences or correlations were identified (data not presented).

Likewise, no significant differences or correlations were observed in relation to the clinical manifestation or sex of patients with paranoid schizophrenia (data not presented).

## Discussion

To our knowledge, this is the first study, which aimed to unite the inflammatory and the neurodegenerational model of paranoid schizophrenia employing recently emerging biomarkers in CSF and serum.

The main finding of the present study indicates that a distinct subgroup of patients with paranoid schizophrenia manifests inflammatory alterations in the CSF, as evidenced by KFLC positivity according to Reiber’s KFLC diagram. In line with the postulations of the inflammatory schizophrenia hypothesis, a subgroup of patients with inflammatory properties indicating an intrathecal humoral immune activation could have been identified^15^. Within this inflammatory subgroup, significantly higher NFL levels in the CSF were observed compared to patients without such inflammation, pointing to a different process of axonal pathology between these subgroups. Moreover, the study indicates a link between more severe illness courses, characterized by increased psychotic episodes and multiple antipsychotic treatment attempts, and increased CSF NFL concentrations, indicating a possible axonal pathology among a subgroup of individuals with paranoid schizophrenia. These findings might also be interpreted as possible link between behavioral-variant frontotemporal dementia and thus as additional biomarker to morphometrical changes as shown by Koutsouleris et al.^7^.

This study represents the first instance of the usage of KFLC interpreted by Reiber´s diagram to identify an inflammatory subset within the population of patients with paranoid schizophrenia, encompassing 20% of patients, despite excluding patients with paranoid schizophrenia with radiological evidence of MS or CIS^27,28^. As consistent with existing literature, KFLC determination exhibited high accuracy in detecting intrathecal immunoglobulin synthesis in patients with non-inflammatory conditions^26^. Nonetheless, it is important to note that KFLC positivity serves as a relatively non-specific indicator for intrathecal inflammation, thus KFLC should be considered as surrogate parameters rather than mediators of inflammatory activity^25^. Nevertheless, both CSF cell analysis via flow cytometry and cyto- and chemokine analyses have indicated inflammatory properties in patients with schizophrenia^10–14^. Interestingly, in the present study, group differences in cyto- and chemokine concentrations were only found, when patients with schizophrenia and MS were compared, with higher concentrations of MCP-1, which was already proposed as important factor within the inflammatory schizophrenia hypothesis^13,15^. However, in contrast to the postulations of this hypothesis, no significant differences between controls and patients with schizophrenia were found^15^.

An additional potential indicator supporting the involvement of inflammatory processes is the finding of elevated NFL levels in CSF among KFLC-positive patients with paranoid schizophrenia compared to their KFLC-negative counterparts. This subgroup appears to experience more pronounced neuroaxonal damage associated with neuro-inflammation than other individuals with paranoid schizophrenia, emphasizing the potential diagnostic value of KFLC determination within the diagnostic evaluation of patients with paranoid schizophrenia. Given that KFLC analysis could identify an inflammatory subset of patients with paranoid schizophrenia characterized by inflammation-related neuro-axonal damage (NFL), it raises questions about the potential benefit of immunomodulatory treatment for these individuals and of the implementation of neuroimaging controls and repeated CSF analyses^11,12,25,29^.

Although elevated CSF NFL levels were observed in KFLC-positive patients with paranoid schizophrenia, NFL concentrations do not appear to hold diagnostic value for all patients with paranoid schizophrenia. This is consistent with existing literature, where most studies highlight NFL measurement as an exclusionary biomarker, aiming to differentiate autoimmune-mediated encephalitis and various forms of dementia from primary psychiatric disorders^17–20^. Runge et al. found that it was not NFL but rather neurofilament medium chain (NFM) that proved effective in distinguishing patients with schizophrenia spectrum disorders from controls^24^. This could potentially clarify the complementary outcomes of two studies that investigated NFL as a biomarker for illness progression and treatment resistance in schizophrenia. On one hand, Eratne et al. reported no differences in plasma NFL concentrations between patients with chronic, treatment-refractory schizophrenia and those without clozapine treatment^22^. On the other hand, Rodrigues-Amorim et al. found significantly increased NFL levels in individuals with chronic schizophrenia compared to healthy controls, with even more pronounced findings within the clozapine-treated, treatment-refractory subgroup characterized by poor prognosis^23^. In line with Rodrigues-Amorim et al., the present study also identified significantly elevated CSF NFL levels among patients with paranoid schizophrenia with a history of multiple antipsychotic treatment attempts and numerous psychotic episodes^23^. However, in accordance with Eratne et al., treatment status at the time of sampling did not lead to significant differences in NFL levels in CSF and serum for patients with schizophrenia^22^.

The current study is not free of limitations. It is limited by the relatively low number of included patients with schizophrenia, which is due to the extensive imaging and laboratory work-up and the exclusion of patients with comorbid psychiatric disorders or other types of schizophrenia, which were mandatory for inclusion. Consequently, only a low number of patients with first-episode psychosis were included, which also limited the possibility of follow-up investigations in the illness course and further sub-analyses according to the episode number, indicating the need of confirmatory studies to validate the findings of this pilot study. The small number of included patients in this pilot study also implicates the inability to adjust for multiple testing. In addition, availability of PANSS (positive and negative syndrome scale) scores in temporal proximity to the CSF sampling would have been important to investigate associations to disease activity and specific symptoms. Nevertheless, relevant conclusions can also be drawn from studies with a relatively low amount of included patients, as shown by Guasp et al. (psychosis *n* = 45), Bavato et al. (schizophrenia *n* = 44) and Rodrigues-Amorim (schizophrenia *n* = 42)^18,21,22^. In addition it has to be considered that the specificity of these findings as well as the generalization of the findings limited since we focused solely on paranoid schizophrenia, without comparing it to other schizophrenia subtypes. Schizophrenia is a heterogeneous disorder with different subtypes exhibiting diverse clinical presentations and possibly distinct biological underpinnings. Further, the present study is partly limited by the effects of antipsychotic medication, since most of the included patients were treated at the time point of sampling and some antipsychotics (olanzapine, clozapine) are known to host anti-inflammatory properties^30–32^. Further, including a broader range of inflammatory markers would have offered more insights into the nature of immune dysregulation in this disorder, since schizophrenia-related inflammation may involve various immune pathways, including the role of microglia, other cytokines and even complement factors^15^. Lastly, the control group used was not constituted from healthy control subjects, but rather neurological patients without evidence of inflammation. While healthy controls would have been the more appropriate control group, the invasive nature of lumbar puncture makes it challenging to recruit such individuals. Patients undergoing elective epidural anesthesia could represent a potential alternative. However, matching this population to our predominantly male study cohort would have been difficult. Prospective biomarker trials in the future should address this issue.

In conclusion, this study highlights a subgroup of patients with paranoid schizophrenia with inflammatory alterations and inflammation-related increased axonal pathology. The significance of these changes and whether they can be influenced by immunomodulatory therapies remains unclear. Further multicenter studies are necessary to elucidate this significance and potentially explore therapeutic options for this subgroup of patients.

## Methods

### Participants and controls

Based on the previous studies of Guasp et al., Bavato et al., and Rodrigues-Amorim et al., a retrospective chart study including a total of 120 patients including 40 individuals who were diagnosed with schizophrenia was carried out within the Cerebrospinal fluid Analysis in Psychiatry (CAP) Consortium^18,21,22^. Due to the pilot study character of this investigation, a sample size estimation or test power analysis was not carried out. Following the approach of previous investigations, as many patients as possible that fulfilled the following inclusion criteria were considered suitable for the present study. Included were all patients with paranoid schizophrenia, which were over 18 years old, for which results of extensive clinical, imaging, serological and CSF analyses were entirely available and which presented to the Department of Psychiatry, Social Psychiatry and Psychotherapy at Hannover Medical School (MHH) between 2007 and 2017. Patients were excluded when the final diagnosis included organic disorders (ICD-10: F06.0, F06.1, F06.2) or substance-induced disorders (ICD-10: F1X.5), or patients diagnosed with comorbid psychiatric illnesses. 5/40 patients with paranoid schizophrenia had to be excluded from the further investigation because neuroimaging revealed inflammatory lesions in typical neuroanatomic regions consistent with multiple sclerosis (MS) or clinically isolated syndrome (CIS), whereas these signs of neuro-inflammation were absent in the remaining 35 patients^27–29^.

Sex and age-matched patients were used as controls. The first inflammatory control group consisted of 35 patients diagnosed with MS according to the 2017 McDonald criteria^29^. The second control group consisted of 40 patients with non-inflammatory disorders (idiopathic intracranial hypertension (IIH) *n* = 10, normal pressure hydrocephalus (NPH) *n* = 10) and patients with symptoms in which no neurological disease was detected (headache *n* = 14, diffuse paresthesia *n* = 6). From patients with IIH and NPH, only the first fraction of CSF, which was also used for routine diagnostic work-up, was used for the present study. None of the control patients (especially of the patients diagnosed with MS) was diagnosed with documented psychiatric symptoms at the time of sampling and manifest psychiatric disorders were not known in the control patient´s medical history. Mean follow-up of 53 months of the patients diagnosed with MS remained also clear of psychiatric diagnoses.

### Diagnostic procedures

A semi-structured interview was conducted according to the German Manual for the Assessment and Documentation of Psychopathology in Psychiatry (AMDP system). PANSS was not available in all patients at a time point close to the time of the sampling and was thus not shown. As part of the routine diagnostic examination, a CSF analysis was performed after lumbar puncture. After extensive diagnostic procedures, a diagnosis of paranoid schizophrenia was made by experienced psychiatrists according to the ICD-10 criteria. All patients with paranoid schizophrenia were examined by magnetic resonance imaging (MRI) and/or computed tomography (CT) and electroencephalogram (EEG).

### Analytical procedures

Analysis of paired CSF and serum samples was performed in the Neurochemical Laboratory of the Department of Neurology of the MHH according to routine diagnostic procedures. Fuchs-Rosenthal counting chambers were used to manually determine the CSF cell count. Kinetic nephelometry (Beckman Coulter IMMAGE, Brea, CA, USA) was used to measure the concentrations of albumin, IgG, IgM, and IgA in CSF and serum samples. Reiber´s quotient diagrams were used to estimate the intrathecally synthesized fraction of IgG, IgA, and IgM^33^. Isoelectric focusing in polyacrylamide gels (EDC, Tübingen, Germany) followed by silver staining was used to detect CSF-specific oligoclonal bands^34^.

### KFLC determination

KFLC concentrations in CSF and serum samples were determined using a nephelometric assay (N Latex FLC kappa Kit; Siemens Healthcare Diagnostics Products GmbH, Erlangen, Germany) according to the manufacturer’s instructions on a BN ProSpec analyzer (Siemens) as described elsewhere^25,26^. The hyperbolic reference range and the amount of intrathecally synthesized KFLC (KFLC IF) was calculated according to the formulas described by Reiber and colleagues (discrimination line: Q_lim_ (FLCk) = (3.27(Q_Alb_^2^ + 33)^0.5^ − 8.2) ×10^− 3^; reference range: Q_mean_ (KFLC) ± 3 CV)^35^. The Reiber diagram was applied to dichotomize whether an intrathecal synthesis of KFLC, which implies an intrathecal inflammatory process, is present or not. When the KFLC IF of a CSF-serum-sample pair in a single patient exceeded Qlim (KFLC IF > 0%), the KFLC concentration in CSF exceeded the amount of KFLC, which might be explained by diffusion from serum into the CSF. Therefore, a KFLC IF > 0% was considered an autochthonous, intrathecal synthesis of KFLC within the CNS in this patient. The relevance of this detection method for intrathecal KFLC synthesis has been validated in different studies, achieving a diagnostic sensitivity for the detection of an intrathecal KFLC synthesis of 92%-100% in patients diagnosed with MS^25^. For statistical comparisons such as group comparisons, the local concentration of KFLC was calculated as follows: KFLC_loc_ = (Q_KFLC_ − Q_mean KFLC_) × KFLC serum (mg/L)^35^. As renal function impairment is known to influence KFLC concentrations in CSF and serum, the estimated glomerular filtration rate (eGFR) was calculated using the CKD-EPI (Chronic Kidney Disease Epidemiology Collaboration) creatinine equations^26,36^. None of the included patients had a reduction in renal function below an eGFR of 70 ml/min/1.73 m².

### NFL determination

CSF and serum NFL were measured using the Simoa technology (Simple Plex Human NF-L Cartridge on an Ella Automated Immunoassay System; Quanterix Corporation, Lexington, MA, USA). The lower limit of detection for NFL was set at 5.4 pg/ml. Samples were diluted according to the manufacturer’s recommendations and concentrations were calculated using the appropriate standard curve.

### Determination of cytokines, chemokines and growth factors

The concentrations of the following cytokines, chemokines and growth factors in CSF were determined by flow cytometry using Legendplex Multiplex assays (BioLegend, London, UK) on a BD FACSCanto II System (Becton Dickinson, Heidelberg, Germany): interleukin (IL)-1β, interferon (IFN)-α2, IFN-γ, tumor necrosis factor (TNF)-α, monocyte chemoattractant protein (MCP)-1, IL-6, IL-8, IL-10, IL-12p70, IL-17 A, IL-18, IL-23, IL-33, visinin like protein (VILIP-) 1, soluble triggering receptor expressed on myeloid cells (sTREM-) 2, brain-derived neurotrophic factor (BDNF), transforming growth factor (TGF-) β1, vascular endothelial growth factor (VEGF), sTREM-1, β nerve growth factor (β-NGF), soluble receptor of advanced glycation end-products (sRAGE), and fractalkine (CX3CL1). Samples were diluted, as recommended by the manufacturer, and concentrations were calculated using the appropriate standard curve. The concentrations of cytokines, chemokines and growth factors in the CSF were only considered for further statistical analysis if detectable concentrations were present in at least 90% of the measured samples.

### Statistical analysis and data visualization

The Shapiro–Wilk test was used to assess for parametric distribution of the decimal variables. Parametric data were described as mean, whereas non-parametric data were described as median, each with the range from the lowest to the highest value (min-max). Group comparison was done using the Wilcoxon Rank sum test for decimal data and the Chi2 test for binary data. For the analysis of paired values the paired t-test (parametrically distributed values) or the Wilcoxon test for paired values (non-parametrically distributed values) was used. When multivariate analyses were performed to verify the independence of the associations with CSF NFL age was considered as covariate. A significant linear regression and correlation emerged between the number of psychotic episodes and CSF NFL concentrations (Fig. 1B). Statistical analysis and creation of figures were performed using SPSS 28.0 (IBM Co., Armonk, New York, USA) and GraphPad Prism (La Jolla, CA, USA; version 5.02).

## Data Availability

The datasets used and/or analysed during the current study are available from the corresponding author on reasonable request.

## References

[1] Jauhar, S., Johnstone, M. & McKenna, P. J. Schizophrenia. *Lancet* **399**(10323), 473–486. 10.1016/S0140-6736(21)01730-X (2022). 10.1016/S0140-6736(21)01730-X 35093231

[2] Sommer, I. E. et al. Early interventions in risk groups for schizophrenia: what are we waiting for? *NPJ Schizophr*. **2**, 16003. 10.1038/npjschz.2016.3 (2016). 27336054 10.1038/npjschz.2016.3 PMC4849435

[3] Kochunov, P. & Hong, L. E. Neurodevelopmental and neurodegenerative models of schizophrenia: white matter at the center stage. *Schizophr Bull.* **40** (4), 721–728. 10.1093/schbul/sbu070 (2014). 24870447 10.1093/schbul/sbu070 PMC4059450

[4] Hoffman, R. E. & Dobscha, S. K. Cortical pruning and the development of schizophrenia: a computer model. *Schizophr Bull.* **15** (3), 477–490. 10.1093/schbul/15.3.477 (1989). 2814376 10.1093/schbul/15.3.477

[5] Kraepelin, E. Dementia praecox and paraphrenia. *J. Nerv. Ment*. **54** (4), 384 (1921).

[6] Kochunov, P. et al. A white matter connection of schizophrenia and alzheimer’s disease. *Schizophr Bull.* **47** (1), 197–206. 10.1093/schbul/sbaa078 (2021). 32681179 10.1093/schbul/sbaa078 PMC7825012

[7] Koutsouleris, N. et al. Exploring links between psychosis and frontotemporal dementia using multimodal machine learning: dementia praecox revisited. *JAMA Psychiatry*. **79** (9), 907–919. 10.1001/jamapsychiatry.2022.2075 (2022). 35921104 10.1001/jamapsychiatry.2022.2075 PMC9350851

[8] Andreou, D. et al. Lower plasma total Tau in adolescent psychosis: involvement of the orbitofrontal cortex. *J. Psychiatr Res.* **144**, 255–261. 10.1016/j.jpsychires.2021.10.031 (2021). 34700214 10.1016/j.jpsychires.2021.10.031

[9] Heneka, M. T. et al. Neuroinflammation in alzheimer’s disease. *Lancet Neurol.* **14** (4), 388–405. 10.1016/S1474-4422(15)70016-5 (2015). 25792098 10.1016/S1474-4422(15)70016-5 PMC5909703

[10] Campana, M. et al. Cerebrospinal fluid pathologies in Schizophrenia-Spectrum Disorder-A retrospective chart review. *Schizophr Bull.* **48** (1), 47–55. 10.1093/schbul/sbab105 (2022). 34480476 10.1093/schbul/sbab105 PMC8781327

[11] Goldsmith, D. R. & Rapaport, M. H. Inflammation and negative symptoms of schizophrenia: implications for reward processing and motivational deficits. *Front. Psychiatry*. **11**, 46. 10.3389/fpsyt.2020.00046 (2020). 32153436 10.3389/fpsyt.2020.00046 PMC7044128

[12] Müller, N. Inflammation in schizophrenia: pathogenetic aspects and therapeutic considerations. *Schizophr Bull.* **44** (5), 973–982. 10.1093/schbul/sby024 (2018). 29648618 10.1093/schbul/sby024 PMC6101562

[13] Räuber, S. et al. Cerebrospinal fluid flow cytometry distinguishes psychosis spectrum disorders from differential diagnoses. *Mol. Psychiatry*. **26** (12), 7661–7670. 10.1038/s41380-021-01244-5 (2021). 34363013 10.1038/s41380-021-01244-5 PMC8873003

[14] Sæther, L. S. et al. Inflammation and cognition in severe mental illness: patterns of covariation and subgroups. *Mol. Psychiatry*. **28** (3), 1284–1292. 10.1038/s41380-022-01924-w (2023). 36577840 10.1038/s41380-022-01924-w PMC10005942

[15] Warren, N. et al. Inflammatory cerebrospinal fluid markers in schizophrenia spectrum disorders: A systematic review and meta-analysis of 69 studies with 5710 participants. *Schizophr Res.* **266**, 24–31. 101016/j.schres.2024.02.001 (2024). 38364730 10.1016/j.schres.2024.02.001

[16] Benkert, P. et al. Serum neurofilament light chain for individual prognostication of disease activity in people with multiple sclerosis: a retrospective modelling and validation study. *Lancet Neurol.* **21** (3), 246–257 (2022). -4422(22)00009 – 6. 35182510 10.1016/S1474-4422(22)00009-6

[17] Al Shweiki, M. R. et al. Neurofilament light chain as a blood biomarker to differentiate psychiatric disorders from behavioural variant frontotemporal dementia. *J. Psychiatr Res.* **113**, 137–140. 10.1016/j.jpsychires.2019.03.019 (2023). 10.1016/j.jpsychires.2019.03.019 30953863

[18] Guasp, M. et al. Neurofilament light chain levels in Anti-NMDAR encephalitis and primary psychiatric psychosis. *Neurology* **98** (14), e1489–e1498. 10.1212/WNL.0000000000200021 (2022). 35145006 10.1212/WNL.0000000000200021

[19] Eratne, D. et al. A pilot study of the utility of cerebrospinal fluid neurofilament light chain in differentiating neurodegenerative from psychiatric disorders: A ‘C-reactive protein’ for psychiatrists and neurologists? *Aust N Z. J. Psychiatry*. **54** (1), 57–67. 10.1177/0004867419857811 (2020). 31220922 10.1177/0004867419857811

[20] Katisko, K. et al. Serum neurofilament light chain is a discriminative biomarker between frontotemporal Lobar degeneration and primary psychiatric disorders. *J. Neurol.* **267** (1), 162–167. 10.1007/s00415-019-09567-8 (2020). 31595378 10.1007/s00415-019-09567-8 PMC6954884

[21] Bavato, F. et al. Altered neuroaxonal integrity in schizophrenia and major depressive disorder assessed with neurofilament light chain in serum. *J. Psychiatr Res.* **140**, 141–148. 10.1016/j.jpsychires.2021.05.072 (2021). 34116440 10.1016/j.jpsychires.2021.05.072

[22] Eratne, D. et al. Plasma neurofilament light chain protein is not increased in treatment-resistant schizophrenia and first-degree relatives. *Aust N Z. J. Psychiatry*. **56** (10), 1295–1305. 10.1177/00048674211058684 (2022). 35179048 10.1177/00048674211058684

[23] Rodrigues-Amorim, D. et al. Plasma β-III tubulin, neurofilament light chain and glial fibrillary acidic protein are associated with neurodegeneration and progression in schizophrenia. *Sci. Rep.* **10** (1), 14271. 10.1038/s41598-020-71060-4 (2020). 32868793 10.1038/s41598-020-71060-4 PMC7459108

[24] Runge, K. et al. Neurodegeneration markers in the cerebrospinal fluid of 100 patients with schizophrenia spectrum disorder. *Schizophr Bull.* **49** (2), 464–473. 10.1093/schbul/sbac135 (2023). 36200879 10.1093/schbul/sbac135 PMC10016411

[25] Konen, F. F. et al. The increasing role of kappa free light chains in the diagnosis of multiple sclerosis. *Cells* **10** (11), 3056. 10.3390/cells10113056 (2021). 34831279 10.3390/cells10113056 PMC8622045

[26] Konen, F. F. et al. Kappa free light chains in cerebrospinal fluid in inflammatory and Non-Inflammatory neurological diseases. *Brain Sci.* **12** (4), 475. 10.3390/brainsci12040475 (2022). 35448006 10.3390/brainsci12040475 PMC9030640

[27] Arneth, B. M. Multiple sclerosis and schizophrenia. *Int. J. Mol. Sci.* **18** (8), 1760. 10.3390/ijms18081760 (2017). 28805697 10.3390/ijms18081760 PMC5578149

[28] Marrie, R. A. et al. Differences in the burden of psychiatric comorbidity in MS vs the general population. *Neurology* **85** (22), 1972–1979. 10.1212/WNL.0000000000002174 (2015). 26519542 10.1212/WNL.0000000000002174 PMC4664123

[29] Thompson, A. J. et al. Diagnosis of multiple sclerosis: 2017 revisions of the McDonald criteria. *Lancet Neurol.* **17** (2), 162–173. 10.1016/S1474-4422(17)30470-2 (2018). 29275977 10.1016/S1474-4422(17)30470-2

[30] Amerio, A. et al. Immunomodulatory effects of clozapine: more than just a side effect in schizophrenia. *Curr. Neuropharmacol.* **22** (7), 1233–1247. 10.2174/1570159X22666231128101725 (2024). 38031778 10.2174/1570159X22666231128101725 PMC10964093

[31] Stamoula, Ε. et al. Atypical antipsychotics in multiple sclerosis: A review of their in vivo Immunomodulatory effects. *Mult Scler. Relat. Disord*. **58**, 103522. 10.1016/j.msard.2022.103522 (2022). 35063906 10.1016/j.msard.2022.103522

[32] Hatziagelaki, E. et al. Effects of olanzapine on cytokine profile and brain-derived neurotrophic factor in drug-naive subjects with first-episode psychosis. *Exp. Ther. Med.* **17** (4), 3071–3076. 10.3892/etm.2019.7285 (2019). 30906479 10.3892/etm.2019.7285 PMC6425240

[33] Reiber, H. Proteins in cerebrospinal fluid and blood: barriers, CSF flow rate and source-related dynamics. *Restor. Neurol. Neurosci.* **21** (3–4), 79–96 (2003). 14530572

[34] Andersson, M. et al. Cerebrospinal fluid in the diagnosis of multiple sclerosis: a consensus report. *J. Neurol. Neurosurg. Psychiatry*. **57** (8), 897–902. 10.1136/jnnp.57.8.897 (1994). 8057110 10.1136/jnnp.57.8.897 PMC1073070

[35] Reiber, H., Zeman, D., Kušnierová, P., Mundwiler, E. & Bernasconi, L. Diagnostic relevance of free light chains in cerebrospinal fluid - The hyperbolic reference range for reliable data interpretation in quotient diagrams. *Clin. Chim. Acta*. **497**, 153–162. 10.1016/j.cca.2019.07.027 (2019). 31351929 10.1016/j.cca.2019.07.027

[32] Levey, A. S. et al. A new equation to estimate glomerular filtration rate. *Ann. Intern. Med.* **150** (9), 604–612. 10.7326/0003-4819-150-9-200905050-00006 (2009). 19414839 10.7326/0003-4819-150-9-200905050-00006 PMC2763564

[36] Konen, F. F. et al. The influence of renal function impairment on kappa free light chains in cerebrospinal fluid. *J. Cent. Nerv. Syst. Dis.* **13**, 11795735211042166. 10.1177/11795735211042166 (2021). 34840504 10.1177/11795735211042166 PMC8619759

